# Lobular carcinoma in situ detectable as a mass on ultrasonography: a case report

**DOI:** 10.1007/s10396-024-01487-z

**Published:** 2024-08-14

**Authors:** Kana Kawanishi, Toshitaka Okuno, Yuki Sakakibara, Kentaro Odani, Satsuki Asai, Yasuhide Kohno, Yoichiro Kuwata

**Affiliations:** 1 Department of Breast Surgery, Kobe City Nishi-Kobe Medical Center, 7-5-1 Koji-Dai, Nishi-Ku, Kobe, 651-2273 Japan; 2 Department of Clinical Laboratory, Kobe City Nishi-Kobe Medical Center, Kobe, Japan; 3 Department of Pathology, Kobe City Nishi-Kobe Medical Center, Kobe, Japan; 4 Department of Diagnostic Radiology, Kobe City Nishi-Kobe Medical Center, Kobe, Japan

## Image-breast

**Fig. 1:**
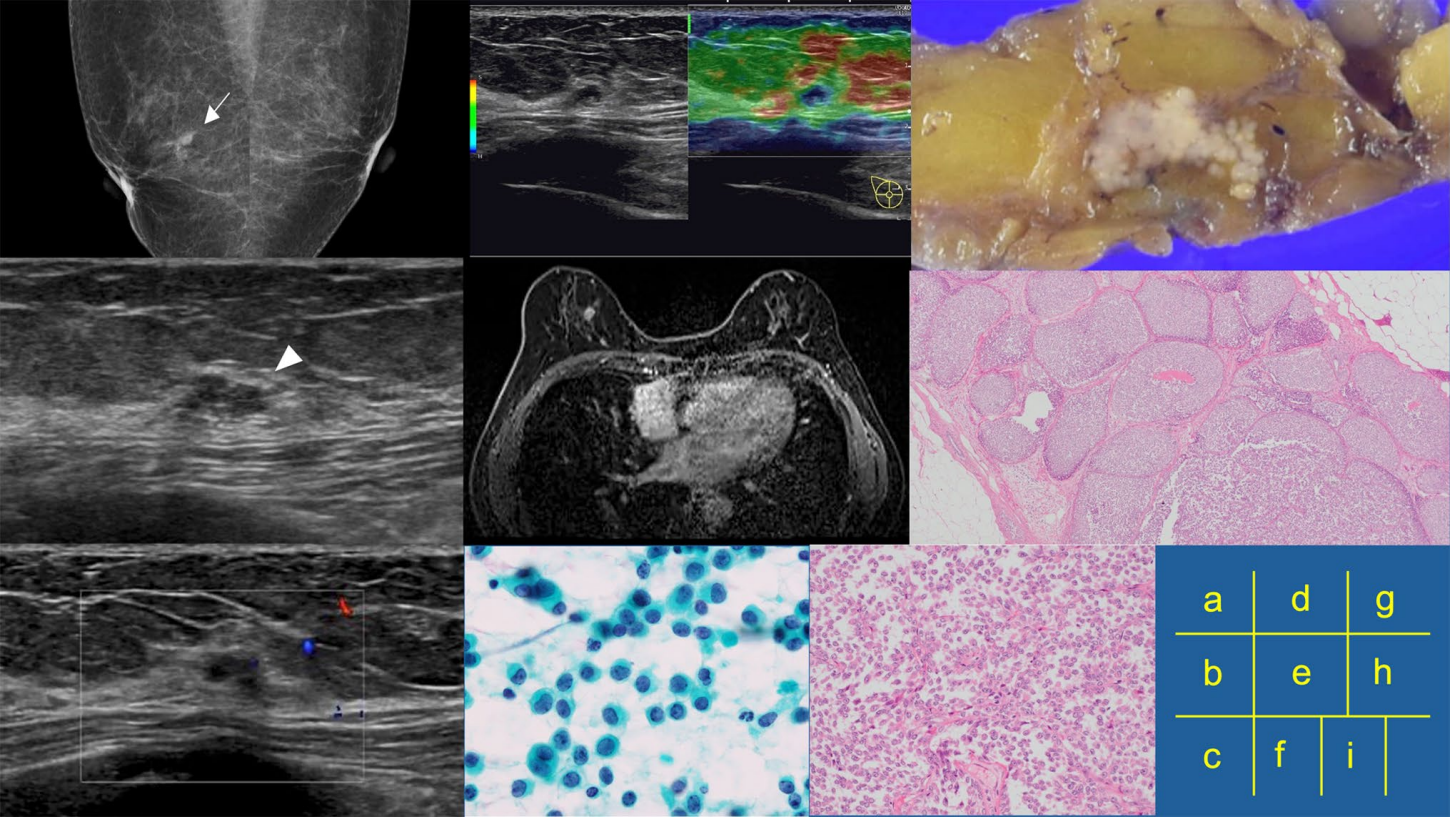
Imaging and pathological findings of lobular carcinoma in situ detectable as a mass on ultrasonography. **a** Mammograms showing a mass without calcification in the upper portion of the right breast (white arrow). **b** B-mode ultrasonography showing a 9-mm hypoechoic, lobulated mass (arrowheads). **c** Color Doppler ultrasonography showing no blood flow signal. **d** Elastography showing decreased elasticity, interpreted as a Tsukuba elasticity score of 4. **e** Magnetic resonance imaging showing a lobulated mass with irregular margins measuring 9 mm in its greatest dimension. **f** Aspiration cytology reveals numerous clusters of atypical cells scattered across a bloody background. The nuclei were small and uniform with increased chromatin and small nucleoli. **g** The tumor is seen as pale clustered nodules on the surgical specimen. **h** Monomorphic, dense proliferation of tumor cells. **i** Poorly cohesive cells with a round shape and inconspicuous cytoplasm are observed

A woman in her 60 s was referred to our department due to a mass detected in the right breast during a screening mammography (Fig. 1a). Ultrasonography revealed a 9-mm hypoechoic, microlobulated mass with posterior enhancement (Fig. 1b). Color Doppler imaging showed no blood flow (Fig. 1c). Elastography showed decreased elasticity, which was interpreted as a Tsukuba elasticity score of 4 (Fig. 1d).

Magnetic resonance imaging (MRI) showed a lobulated mass with irregular margins. The mass, uniformly contrast-enhanced, displayed a fast washout pattern (Fig. 1e). We diagnosed the lesion as BI-RADS category 4A, presuming a benign breast lesion such as fibrocystic change or fibroadenoma.

Aspiration cytology revealed numerous clustered atypical cells scattered across a bloody background. The nuclei were small and uniform with increased chromatin and some small nucleoli, and interpreted as malignant (Fig. 1f). She preferred surgery over core needle biopsy; therefore, we performed a partial mastectomy and sentinel lymph node biopsy. On the cut surface, the tumor appeared as pale clustered nodules (Fig. 1g) with a monomorphic, dense proliferation of dyscohesive round cells with an inconspicuous cytoplasm (Figs. 1h and i). No ductal structure or benign breast changes were observed. Small, round nuclei, located centrally with smooth nuclear membranes and inconspicuous nucleoli, were diagnosed as classic lobular carcinoma in situ (LCIS).

LCIS is both a risk factor and a nonobligate precursor of breast carcinomas. Foote and Stewart first described LCIS in 1941 and reported that it is an incidental microscopic finding. The clinical diagnosis of LCIS remains elusive [1], with < 2% of classic LCIS cases warranting biopsy due to the associated imaging abnormalities, including grouped amorphous or granular calcifications on mammography or heterogeneous non-mass-like enhancement on MRI [2]. Coi et al. reported that the common ultrasonographic findings of LCIS were irregular shape, ill-defined margins, and hypoechogenicity [3].

LCIS is a proliferation of atypical monotonous epithelial cells that fill and distend the acinar units of the lobule [4]. The ultrasonographic findings in our patient showed a small, lobulated shape, poor blood flow, and decreased elasticity, reflecting the histological findings of tumor cell-filled foci with no prominent blood vessels in the surrounding stroma. And arising in an elderly woman with non-dense breast tissue, this case exhibited a small lobulated mass image reflecting the pathological features.

This case underscores LCIS detectability through mammography, ultrasonography, and MRI. Recognizing these imaging features proves valuable in prompting image-guided biopsy and facilitating an accurate diagnosis of LCIS.
